# Efficacy of Olfactory and Pareidolia Tests Compared With That of Indicative Biomarkers in Diagnosis of Dementia With Lewy Bodies

**DOI:** 10.3389/fneur.2020.540291

**Published:** 2020-09-11

**Authors:** Yuta Inagawa, Hidekazu Kanetaka, Akito Tsugawa, Shu Sakurai, Shuntaro Serisawa, Soichiro Shimizu, Hirofumi Sakurai, Haruo Hanyu

**Affiliations:** Department of Geriatric Medicine, Tokyo Medical University, Tokyo, Japan

**Keywords:** MIBG cardiac scintigraphy, DAT (dopamine transporter), pareidolia test, OSIT-J, AD, DLB, dementia with Lewy bodies

## Abstract

**Purpose:** Although olfactory decline and visual hallucinations are useful in distinguishing dementia with Lewy bodies (DLB) from Alzheimer's disease (AD) in a clinical setting, neither is easy to evaluate objectively. The pareidolia test is used to assess susceptibility to visual hallucinations, while in Japan, the Odor Stick Identification Test for the Japanese (OSIT-J) is used to objectively quantify olfactory decline. The present study investigated the efficacy of these olfactory and pareidolia tests in differentiating AD from DLB. Their usefulness was then compared with that of the indicative biomarkers in neuroimaging for a clinical diagnosis of DLB listed in the Fourth Consensus Report of the Dementia with Lewy Bodies Consortium.

**Methods:** A total of 24 probable DLB and 22 probable AD patients were enrolled. All underwent 4 diagnostic procedures: uptake of dopamine transporter in single photon emission computed tomography (DaT-SPECT) and meta-iodobenzylguanidine (MIBG) in myocardial scintigraphy, the pareidolia test, and OSIT-J. The sensitivity, specificity, and accuracy of these methods in differentiating DLB from AD were compared.

**Results:** Sensitivity and specificity in differentiating DLB from AD were 86 and 100% by the heart-to-mediastinum ratio of MIBG uptake; 82 and 96% by the specific binding ratio on DaT-SPECT; 77 and 67% by the combination of OSIT-J and pareidolia test scores; 73 and 62% by the pareidolia test scores; and 77 and 58% by the OSIT-J scores, respectively.

**Conclusions:** The present results suggest that the pareidolia and OSIT-J tests may be considered before resorting to nuclear neuroimaging in the diagnosis of DLB.

## Introduction

Dementia with Lewy bodies (DLB) is recognized as the second most common cause of degenerative dementia in older people after Alzheimer's disease (AD). The Fourth Consensus Report of the Dementia with Lewy Bodies Consortium, published in 2017 (1), lists two indicative biomarkers of DLB in neuroimaging: uptake of dopamine transporter in single photon emission computed tomography (DaT-SPECT) and that of meta-iodobenzylguanidine (MIBG) in myocardial scintigraphy.

These same diagnostic criteria also include visual hallucinations as a core clinical feature of DLB, along with olfactory decline as a supportive clinical feature that is often also present in such cases. This latter, however, has yet to be proven diagnostically specific. Although these two symptoms are useful in distinguishing DLB from AD in a clinical setting, however, neither is easy to evaluate quantitively.

Pareidolia is similar to visual hallucinations and may be an alternative indicator of hallucinations. The pareidolia test was developed as a tool for evaluating pareidolia (2). The Odor Stick Identification Test for the Japanese (OSIT-J) was developed as a tool for objectively assessing olfactory decline in Japanese people. It is easy to perform and involves the use of odorants and cards (3). The purpose of the present study was to determine the efficacy of olfactory and pareidolia tests in differentiating between AD and DLB. Their usefulness was then compared with that of the indicative biomarkers in neuroimaging for a clinical diagnosis of DLB listed in the 2017 report described above. To our knowledge, this is the first study to compare the diagnostic value of the pareidolia test, OSIT-J, DaT-SPECT, and MIBG myocardial scintigraphy in differentiating DLB from AD in a patient group that underwent all 4 procedures.

## Methods

### Patients

A total of 24 probable DLB and 22 probable AD patients who underwent 4 diagnostic procedures (DaT-SPECT, MIBG, the pareidolia test, and OSIT-J®) on their first visit to our establishment were enrolled from the Memory Disorder Clinic at the Department of Geriatric Medicine, Tokyo Medical University. The diagnosis of probable DLB was based solely on the core clinical features of DLB listed in the 2017 report discussed above (1). The diagnosis of probable AD was based on the criteria established by the National Institute on Aging-Alzheimer's Association in 2011 (4). All the AD patients enrolled in this study were of the typical amnestic subtype, and all presented with the complaint of impaired memory. All patients falling under other clinical subtypes of AD (those with posterior cortical atrophy, of the aphasia subtype, or with the frontal variant, for example) were deemed ineligible for inclusion.

The severity of dementia was classified as 0.5 (questionable) to 2 (moderate) in both groups of patients based on Clinical Dementia Rating (5). The Mini-Mental State Examination (6) scores were between 12 and 29.

None of the patients had a history of cerebrovascular disease, degenerative disease, infarction in the region of the basal ganglia or intracranial lesions on brain MRI, thyroid disease, diabetes mellitus, or previous relevant cardiac disease; none were taking any medications or substances known to interact with the striatal binding of ^123^I-2β-Carbomethoxy-3β-(4-iodophenyl)-N-(3-fluoropropyl) nortropane (^123^I-FP-CIT) (e.g., cocaine, amphetamines, bupropion, selective serotonin reuptake inhibitors) (7, 8) or that might affect accumulation of MIBG (9). The exclusion criteria comprised a history of nasal disease and current smokers and extremely poor visual acuity, as these may have compromised the reliability of the results of the OSIT- J and pareidolia tests.

The protocol of this study was approved by the Ethics Committee of Tokyo Medical University. Informed consent was obtained from all the study participants (either the patients themselves or their closest relative) before entry, following a detailed explanation of the aim of the study.

### Dopamine Transporter Uptake in Single Photon Emission Computed Tomography

Three hours after injecting approximately 185 MBq of ^123^I-FP-CIT, projection data were acquired on a 128 × 128 matrix by a Siemens Symbia T16 equipped with a low-to-medium energy general purpose collimator. The specific binding ratio (SBR) was calculated semi-quantitatively using DAT VIEW software (AZE, Tokyo, Japan) based on the Tossici-Bolt method described previously (10). In the present study, the left-right average of the SBR was used for the analysis.

### Meta-Iodobenzylguanidine Myocardial Scintigraphy

A total of 123 MBq of ^123^I-MIBG was administered intravenously. Early and delayed SPECT were performed at 20 min and at 4 h after injection, respectively. Planar scanning and SPECT were performed with a gamma camera (PRISM 2000VP, Picker). After scatter correction, relative organ uptake was determined by setting the anterior region of interest (ROI) (11). The heart-to-mediastinum ratio (H/M) was calculated by dividing the left ventricular ROI count density by the mediastinal ROI count density according to the standard method (12). Delayed phase image data were used for the analysis.

### Pareidolia Test

The pareidolia test was used to determine the presence of hallucinations, a core clinical feature of DLB. The face version of the noise pareidolia test developed by Yokoi et al. (Tohoku University, Japan) was adopted (13). A total of 40 black and white images (16 × 16 cm^2^) with a spatial frequency of 1/f^3^ are used in this test, in 8 of which a face is included. All the patients were allowed to undergo training in this test prior to the recording of the results. They were instructed to state whether they observed a face or not on presentation of each image. When the participants observed a face, they were requested to point to it.

The pareidolic illusion rate is defined as the ratio of the total number of images (40) to the number of images that are mistakenly recognized as containing the image of a face (13).

### Odor Stick Identification Test (OSIT-J)

Decline in olfactory function was determined with the OSIT-J (Daiichi Yakuhin, Co., Tokyo, Japan). This test comprises the following 12 kinds of odorant: rose, condensed milk, Japanese orange, curry, roasted garlic, fermented beans, sweaty socks, cooking gas, menthol, India ink, wood, and Japanese cypress (*hinoki*), all of which are familiar to Japanese people (14).

Each odorant is encapsulated in a melamine resin microcapsule, which resembles a stick of lip gloss. The examiner uses each one to paint a 2-cm circle on a piece of paper. They then crush the microcapsule by folding the piece of paper and handing it to the patient. After sampling each odor, the patient is presented with a card showing the names of the 4 odors and asked to make selection. The total number of correct answers for the 12 odorants presented is the OSIT-J score, and the range of the OSIT-J score is 0–12 (15). The cut-off value for decreased olfaction in elderly people over the age of 60 yr is 4–6 points (16).

### Statistical Analysis

The Mann-Whitney test, χ2 test, and Student's *t*-test were used for the analysis. All the values obtained are expressed as the mean ± SD or median, min-max. These data were statistically analyzed using IBM SPSS Statistics version 25 software (Chicago, IL).

The sensitivity and specificity of the respective diagnostic index (H/M ratios of MIBG uptake in the delayed phase); the left-right average of the SBR on DaT-SPECT; the pareidolic illusion rate; the OSIT-J® score; and the combined pareidolic illusion rate and OSIT-J® score to differentiate between DLB and AD were assessed using receiver-operating characteristic (ROC) analysis.

The method of DeLong was used to analyze differences in the area under the curve (AUC) for each result. These data were statistically analyzed using MedCalc version 19 software (Belgium).

A *p* < 0.05 was considered to indicate a statistically significant difference between the two groups.

For the combined use of the pareidolia test and OSIT-J® score, we have developed the “OSIT-J^*^Pareidolia Test” index, which is defined as the OSIT-J® score^*^ (1-pareidolic illusion rate).

## Results

Table 1 shows the characteristics of the patients. The mean age of the patients in the DLB group was 82.4 ± 5.0 yr, while that in the AD group was 80.0 ± 5.6 yr. No significant difference was observed between the two groups (*p*-value, 0.071).

**Table 1:** Patient characteristics.

	**DLB group**	**AD group**	***p*-value**
Number of patients	24	22	–
Age (year) (median, min–max.)	82.4 (73–91)	80.5 (61–88)	0.071^a^
Male/Female	14/10	11/11	0.5716
Educational history(year) (mean, SD)	13.1 ± 2.8	12.1 ± 2.8	0.227^c^
MMSE score (median, min–max.)	22.9 ± 3.4	20.4 ± 4.8	0.046^a^
OSIT—J score (median, min—max.)	2.0 (0–8)	4.0 (0–11)	0.004^a^
The pareidolic illusion rate (%) (mean, SD)	10.6 + 11.7	3.7 ± 6.6	0.011^c^
OSIT—J*Pareidolia test (median, min–max.)	1.8 (0.0–6.8)	3.9 (0.0–11.0)	0.003^a^
DaT—SPECT SBR(average) (median, min–max.)	2.2 (0.1–4.2)	4.5 (2.7–7.4)	<0.001^a^
MIBG H/M ratio (delay phase) (median, min–max.)	1.2 (0.9–2.2)	3.0 (1.2–4.1)	<0.001^a^

a *Mann-Whitney U-test*.

b *X^2^ test*.

c *Student's t-test*.

The H/M ratios of MIBG uptake in the delayed phase were 1.3 ± 0.3 and 2.9 ± 0.7 (*p* < 0.001); the left-right average of the SBR on DaT-SPECT 2.2 ± 1.2 and 4.7 ± 1.2 (*p* < 0.001); the OSIT-J^*^ Pareidolia Test scores 2.3 ± 1.9 and 4.2 ± 2.6 (*p* < 0.003); the pareidolia test scores 10.6 ± 11.7 and 3.7 ± 6.6 (*p* < 0.011); and the OSIT-J scores 2.5 ± 2.0 and 4.4 ± 2.6 (*p* < 0.004) in the DLB and AD patients, respectively. The results of all the tests administered (MIBG myocardial scintigraphy, DaT-SPECT, OSIT-J^*^ Pareidolia combination test, pareidolia test, and OSIT-J) all showed a significant difference between the AD and DLB groups.

**Figure 1:**
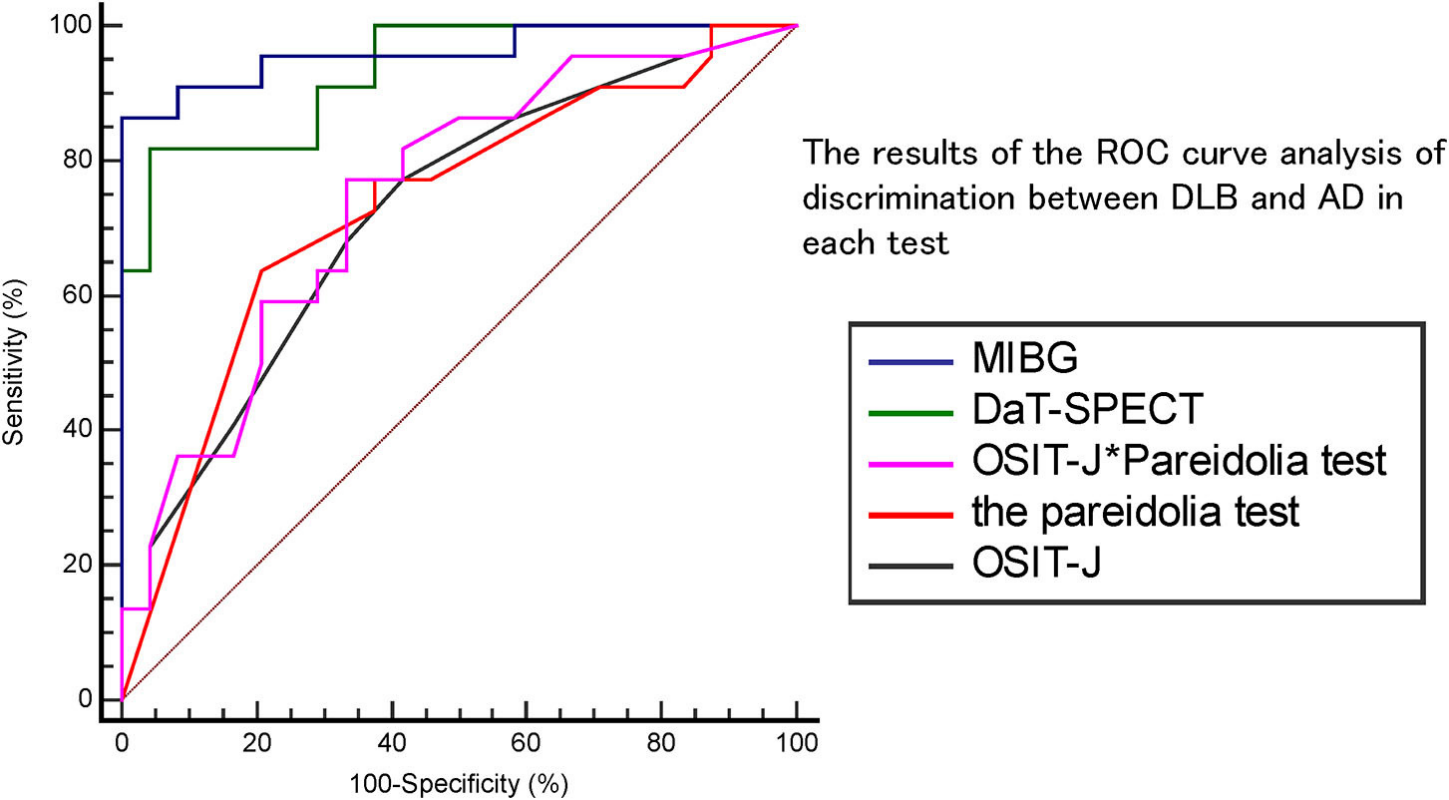
The results of the ROC curve analysis of discrimination between DLB and AD in each test.

Figure 1 and Table 2 show the results of the ROC curve analysis of discrimination between DLB and AD in each test. With MIBG myocardial scintigraphy, the AUC was 0.96, the cut-off score 2.54, sensitivity 86%, specificity 100%, positive predictive value (PPV) 100%, and negative predictive value (NPV) 67%; DaT-SPECT showed an AUC of 0.93, cut-off score of 3.93, sensitivity of 82%, specificity of 96%, PPV of 85%, and NPV of 95%; the OSIT-J^*^ Pareidolia Test showed an AUC of 0.75, a cut-off score of 2.93, sensitivity of 77%, specificity of 67%, PPV of 73%, and NPV of 67%; the pareidolia test showed an AUC of 0.73, cut-off score of 2.50, sensitivity of 73%, specificity of 62%, PPV of 70%, and NPV of 74%; and the OSIT-J showed an AUC of 0.72, cut-off score of 3, sensitivity of 77%, specificity of 58%, PPV of 74%, and NPV 63%. Here, a reduction in the OSIT-J® score was defined as 3 points or fewer, and pareidolia-positive was defined as having a pareidolic illusion rate of >2.5%.

**Table 2:** The results of the ROC curve analysis of discrimination between DLB and AD in each test.

	**Sensitivity (%)**	**Specificity (%)**	**PPV (%)**	**NPV (%)**	**Cut-off score**	**AUC**
MIBG	86	100	100	67	2.54	0.96
DaT-SPECT	82	96	85	95	3.93	0.93
OSIT-J*Pareidolia test	77	67	73	67	2.93	0.75
The pareidolia test	73	62	70	74	2.50	0.73
OSIT-J	77	58	74	63	3	0.72

*PPV, Positive Predictive Value; NPV, Negative Predictive Value; AUC, Area under the curve*.

Table 3 shows the results of the comparison of the two ROC curves. The *p*-value for DaT-SPECT vs. MIBG myocardial scintigraphy was 0.54; DaT-SPECT vs. OSIT-J, 0.001; DaT-SPECT vs. the pareidolia test, 0.02; DaT-SPECT vs. the OSIT-J^*^ Pareidolia Test, 0.02; MIBG myocardial scintigraphy vs. OSIT-J, 0.001; MIBG myocardial scintigraphy vs. the pareidolia test, 0.005; MIBG myocardial scintigraphy vs. the OSIT-J^*^ Pareidolia Test, 0.002; OSIT-J vs. the pareidolia test, 0.94; OSIT-J vs. the OSIT-J^*^ Pareidolia Test, 0.75; and the pareidolia test vs. the OSIT-J^*^ Pareidolia Test, 0.9.

**Table 3:** The results of the ROC curve analysis of discrimination between DLB and AD in each test.

	**Difference between areas**	**Standard Error**	**Z statistic**	***p*-value**
MIBG vs. DaT-SPECT	0.028	0.046	0.613	0.540
MIBG vs. OSIT-J	0.237	0.072	3.294	0.001
MIBG vs. the pareidolia test	0.228	0.081	2.832	0.005
MIBG vs. OSIT-J*Pareidolia test	0.214	0.070	3.073	0.002
DaT-SPECT vs. OSIT-J	0.208	0.083	2.520	0.011
DaT-SPECT vs. the pareidolia test	0.200	0.087	2.304	0.021
DaT-SPECT vs. OSIT-J*Pareidolia test	0.186	0.083	2.247	0.024
OSIT-J vs. the pareidolia test	0.008	0.109	0.078	0.938
OSIT-J vs. OSIT-J*Pareidolia test	0.023	0.013	1.703	0.089
the pareidolia test vs. OSIT-J*Pareidolia test	0.014	0.101	0.140	0.089

*PPV, Positive Predictive Value; NPV, Negative Predictive Value; AUC, Area under the curve*.

## Discussion

Although both DaT-SPECT and MIBG myocardial scintigraphy are neuroimaging techniques that have high power in diagnosing DLB, they have the disadvantage of being expensive, and can be performed only at hospitals with the necessary equipment. Therefore, there is a growing need for biomarkers that can be tested more easily and cheaply. Typical subjective symptoms related to the diagnosis of DLB include visual hallucinations and decreased olfaction. Assessment of visual hallucinations and decreased olfaction is difficult, however, especially when the patient's own awareness of these symptoms is poor, or when the caregiver is unable to grasp the true situation as the patient is living alone. The question also remains as to whether subjective reporting of symptoms is truly reliable. Therefore, we hypothesized that if we could objectively assess visual hallucinations and decreased olfaction, it might be useful as a biomarker for DLB diagnosis. The OSIT-J and pareidolia tests are covered by the initial cost of consultation, relieving the patient of a significant financial burden. Moreover, they can be performed in clinics or other small facilities, as no special equipment or space is required.

The results of the present study revealed that patients in the DLB group had a significantly higher pareidolic illusion rate and lower OSIT-J score than those in the AD group. These findings indicate that pareidolia tests, which assess susceptibility to visual hallucinations, and the OSIT-J, which allows quantitative assessment of olfactory decline, are useful in differentiating DLB from AD.

Although the pareidolia test did not show such high sensitivity and specificity in present study, one previous study reported a sensitivity of 100% and specificity of 88% in differentiating DLB from AD (2). This previous study showed no significant difference in visual acuity between the DLB and AD groups. The fact that the current study did not perform an accurate visual acuity test and only excluded patients who were noted to have obvious vision loss during the neuropsychological examination may have affected the accuracy of the pareidolia test.

It has been reported that hyposmia was not only pre-symptomatic of DLB, but also more frequent than the core clinical features normally associated with DLB (17). Therefore, sense of smell is widely considered a major factor in the diagnosis of DLB, making its objective evaluation by means of the OSIT-J® all the more important. In the present study, the OSIT-J® cut-off score for distinguishing DLB from AD was 3 points. One earlier study suggested that the cut-off value for decreased olfaction in elderly people over the age of 60 years is 4–6 points (16). In brief, the cut-off value for discriminating DLB from AD with OSIT-J® was lower than that in screening for hyposmia in the elderly. One study noted that the sense of smell was reduced in not only DLB, but also AD patients (18). Therefore, we believe that the cut-off value in discriminating AD from DLB to be lower than that for olfactory decline.

The diagnostic utility of the tests applied in the present study was not as high as that of DaT-SPECT or MIBG myocardial scintigraphy, both of which have been recommended as useful in detecting indicative biomarkers of DLB in recent diagnostic criteria. Nonetheless, we believe that they do have some utility as they are cheaper and simpler than these standard alternatives. This suggests that the pareidolia and OSIT-J tests may be useful in determining whether to perform further nuclear imaging, or may aid in the diagnosis in areas where nuclear imaging is not available. The present results also revealed that the discriminatory power of the combination of the olfactory and pareidolia tests was not significantly greater than that with either alone.

Therefore, while it is desirable to perform both tests, only one of them may be useful in some cases. In other words, in obtaining diagnostic clues, only olfactory tests may be suitable for patients with visual impairment and pareidolia tests for those with olfactory disorders.

This study had several critical limitations. Firstly, determination of visual hallucinations was based solely on patient-reported symptoms or third-party reporting (caregiver). Further study should use the scale for visual hallucinations as recommended in the “DIAMOND Lewy study (19).” Secondly, it was carried out at a single memory disorder clinic; therefore, the number of patients enrolled in each group was relatively small. Thirdly, the OSIT-J^*^ Pareidolia Test index is an original index devised by our group for this study. Therefore, statistical weighting was not taken into account when setting this index. Further study is necessary to determine the validity of the OSIT-J^*^ Pareidolia Index. Another potential limitation of the present study was the lack of autopsy confirmation in all cases. Rigorous standardized sets of diagnostic criteria were applied, however, all of which have been shown to have a positive predictive value of >80% when judged by postmortem diagnosis (20, 21). Further, large-scale, multicenter studies taking the results of pathological examination into consideration are required to confirm the present results.

In conclusion, the results of the present study suggest that the pareidolia and OSIT-J® tests may be considered before resorting to nuclear neuroimaging in the diagnosis of DLB.

## Data Availability Statement

The original contributions presented in the study are included in the article/supplementary material, further inquiries can be directed to the corresponding author/s.

## Ethics Statement

The studies involving human participants were reviewed and approved by the Ethics Committee of Tokyo Medical University. The patients/participants provided their written informed consent to participate in this study.

## Author Contributions

YI and HK: conception and design of the study. SSa, SSe, HK, and SSh: acquisition and analysis of data. HS and HH: drafting a significant portion of the manuscript or figures. All authors contributed to the article and approved the submitted version.

## Conflict of Interest

The authors declare that the research was conducted in the absence of any commercial or financial relationships that could be construed as a potential conflict of interest.
